# The Drosophila Gap Gene Network Is Composed of Two Parallel Toggle Switches

**DOI:** 10.1371/journal.pone.0021145

**Published:** 2011-07-01

**Authors:** Dmitri Papatsenko, Michael Levine

**Affiliations:** 1 Department of Gene and Cell Medicine, Mount Sinai School of Medicine, Black Family Stem Cell Institute, New York, New York, United States of America; 2 Department of Molecular and Cell Biology, University of California, Berkeley, California, United States of America; University of South Florida College of Medicine, United States of America

## Abstract

Drosophila “gap” genes provide the first response to maternal gradients in the early fly embryo. Gap genes are expressed in a series of broad bands across the embryo during first hours of development. The gene network controlling the gap gene expression patterns includes inputs from maternal gradients and mutual repression between the gap genes themselves. In this study we propose a modular design for the gap gene network, involving two relatively independent network domains. The core of each network domain includes a toggle switch corresponding to a pair of mutually repressive gap genes, operated in space by maternal inputs. The toggle switches present in the gap network are evocative of the phage lambda switch, but they are operated positionally (in space) by the maternal gradients, so the synthesis rates for the competing components change along the embryo anterior-posterior axis. Dynamic model, constructed based on the proposed principle, with elements of fractional site occupancy, required 5–7 parameters to fit quantitative spatial expression data for gap gradients. The identified model solutions (parameter combinations) reproduced major dynamic features of the gap gradient system and explained gap expression in a variety of segmentation mutants.

## Introduction

Fertilized eggs of *Drosophila* contain several spatially distributed maternal determinants - morphogen gradients, initiating spatial patterning of the embryo. One of the first steps of *Drosophila* embryogenesis is the formation of several broad gap gene expression patterns within first 2 hrs of development. Gap genes are regulated by the maternal gradients, so their expression appears to be hardwired to the spatial (positional) cues provided by the maternal gradients [1]; in addition, gap genes are involved into mutual repression [2]. How the maternal positional cues and the mutual repression contribute to the formation of the gap stripes has been a subject of active discussion [3], [4], [5].

Accumulated genetics evidence and results of quantitative modeling suggest the occurrence of maternal positional cues (position-specific activation potentials), contributing to spatial expression of four trunk gap genes: *knirps* (*kni)*, *Kruppel* (*Kr*), *hunchback* (*hb*) and *giant* (*gt*). Existing data suggest that the central Knirps domain stripe is largely the result of activation by Bicoid (Bcd) and repression by Hunchback [1], [4], [6]. Central domain Kruppel stripe is the result of both activation and repression from Hunchback, which acts as a dual transcriptional regulator on *Kr* [5], [7], [8]. Hunchback is one of the most intriguing among the segmentation genes. Maternal *hb* mRNA is deposited uniformly, but its translation is limited to the anterior, zygotic anterior expression of *hb* is under control of Bcd and Hb itself [9], [10], [11], [12]. Zygotic posterior expression of Hunchback (not included in the current model) is under the control of the terminal *torso* signaling system [13]. Giant is activated by opposing gradients of Bicoid and Caudal and initially exprxessed in a broad domain, which refines later into anterior and posterior stripes. This late pattern appears to be the consequence of Kruppel repression [2], [14].

Predicting functional properties of a gene network combining even a dozen genes may be a difficult task. To facilitate the functional exploration, gene regulatory networks are often split into network domains or smaller units, network motifs with known or predictable properties [15], [16], [17]. The network motif based models can explain dynamics of developmental gradients [18] and even evolution of gradient systems and underlying gene regulatory networks [19]. The gene network leading to the formation of spatial “gap” gene expression patterns is an example, where simple logic appeared to be far behind the system’s complexity [6], [20]. Gap genes provide first response to maternal gradients in the early fly embryo and form a series of broad stripes of gene expression in the first hours of the embryo development. While the system has been extensively studied in the past two decades both *in vivo* [2], [21], [22] and *in silico* [5], [6], [23] a simple and comprehensive model explaining function of the entire network has been missing [24], [25].

In the current study, a modular design has been proposed for the gap gene network; the network has been represented as two similar parallel modules (or two sub networks). Each module involved three network motifs, two for maternal inputs (one for one gap gene) and a toggle switch describing mutual repression in the pair of the gap genes. Formally, the toggle switches present in the gap gene network are evocative of the bistable phage lambda switch [26], [27]; however, they are operated by maternal inputs and their steady state solutions depend on spatial position in embryo, not environmental variables. The proposed modular design accommodated 5–7 realistic parameters and reproduced major known features of the gap gene network.

## Results and Discussion

### 1. General model for a toggle switch with variable synthesis rates

**Figure 1:**
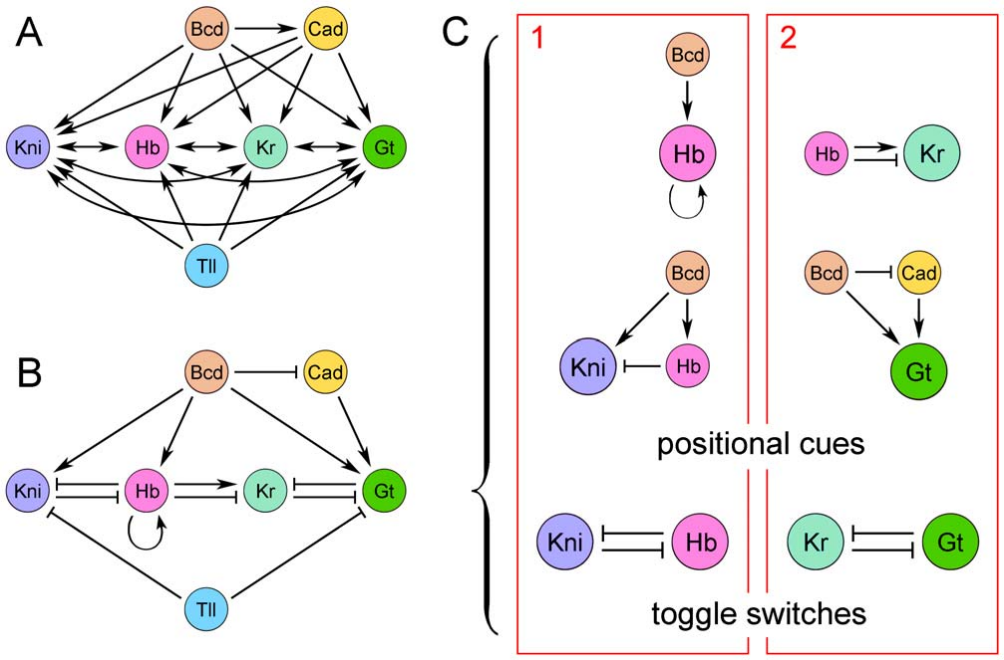
Architecture of the gap gene network. (A) A “connectionist” network starts from total connectivity. Fitting connectionist model to data removes unnecessary links and adds signs (activation or repression). (B) A “minimalist” network, connectivity reflects critical interactions supported by genetic data and connectionist models. (C) Modular design: the minimalist network is split into parallel sub networks (1 and 2, in red), each containing one toggle switch, operated by two positional cues.

Mutual repression between the gap genes represents a critical component of the gap gene network. However, not all possible repressive interactions between the four trunk gap genes are equally important. Analysis of expression in gap gene mutants and exploration of connectionist models (see **Figure 1A**) provided evidence for mutually repressive interactions between *giant* and *Kruppel* [2], [14] as well as *hunchback* and *knirps* [1], [6]. The pairs Gt-Kr and Hb-Kni have partially overlapping expression patterns. With the account of strongest repressive interactions [23] (see **Figure 1B**), the mutually repressive gap gene pairs Gt-Kr and Hb-Kni can be considered as two parallel toggle switches operated by the maternal positional cues (**Figure 1C**).

**Figure 2:**
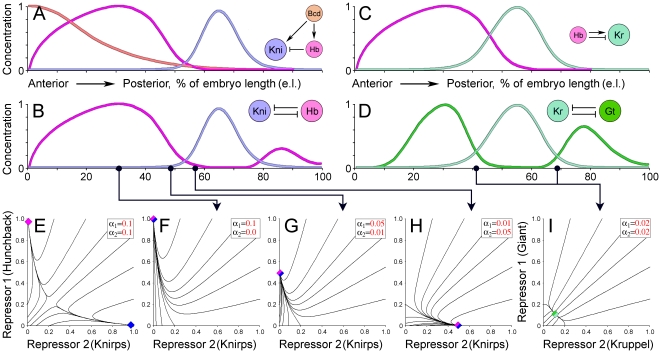
Positionally operated toggle switches. (A) Gradients for Bicoid (Bcd) and Hunchback (Hb) define positional potential (cue) for Knirps. (B) Relative distribution of mutually repressive Hb and Kni gradients. (C) Dual regulation by Hb defines positional cue for Kr. (D) Relative distribution of mutually repressive Giant (Gt) and Kruppel (Kr) gradients is similar to that of Hb and Kni (parallel module). (E) Phase portrait of a bistable toggle switch; the system has two dynamic attractors (diamonds) if rates of synthesis *α*_1_ and *α*_2_ (in red) are high. (F–I) Predicted behavior of a simulated toggle switch is in agreement (see the arrows) with the observed distribution of gap gradients in Hb-Kni and Gt-Kr pairs. If the synthesis rates *α* (positional cues) are asymmetric or low at a given coordinate, the toggle switch has a single attractor at that position.

A positionally operated toggle switch can be simulated by variation of synthesis rates for repressors (see methods, eq. 12). In this case, the synthesis rates are analogous to maternal inputs, changing along the anterior-posterior axis of the embryo (**see** **Figure 2A, C**). If the synthesis rates are high for both repressors, then the system resembles classical bistable phage lambda switch with two dynamic attractors [26], [27] (**Figure 2E**). If the synthesis rates are asymmetric or low, then the system has only one dynamic attractor (monostability) and a single solution (**Figure 2F–I**). The observed ratios of concentrations in the simulated positionally operated toggle switch are in a good agreement with ratios observed between the mutually repressive gradients Hb-Kni and Gt-Kr (**Figure 2B, D**). For instance, zygotic expression of *hb* in the anterior is driven by Bicoid acting on *hb* P2 promoter, containing an array of moderate-affinity binding sites for Bcd, responding to high and intermediate Bcd concentrations [10]. The *knirps cis* enhancer contains cooperative arrays of high-affinity Bcd sites that are sensitive to lower Bicoid concentrations [28]. Expression of *kni* is excluded from the anterior domain of Hb due to Hb repression, which correspond to asymmetric synthesis rates (*α*_Hb_ >> *α*_Kni_) at around 30% of embryo length (e.l.) and elimination of Kni. Instead, at around 65% of e.l. synthesis of Hb is lower due to low sensitivity of P2 promoter to Bicoid, while *kni cis* enhancer with its high affinity Bcd sites is still active; this corresponds to a reverse ratio of the synthesis rates (*α*_Hb_ << *α*_Kni_) and elimination of Hb. The simulated toggle switch also produced solutions corresponding to equally low concentrations of repressors, as shown on the example of Gt-Kr pair of gradients (**Figure 2I**).

### 2. Steady-state models describing maternal positional cues for the trunk gap genes

The positional cues determining the variable synthesis rates are established by maternal inputs. Most of these positional signals are known from experimental or *in silico* analysis of the gap gene network [2], [5], [6], [21], [22], [23]. Below is a formal summary of the maternal inputs, expressed via steady-state fractional site occupancy models (see methods section) [4], [17], [29], [30], [31] for the four trunk gap genes. Full versions of the models are available in Supporting Information File S1, on page 2 “Detailed models describing positional cues for maternal and gap genes”.

#### Hunchback

Bicoid and Hunchback itself regulate expression of *hunchback*; both regulators are required (operator AND) for Hunchback expression:



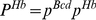
(1)Substitution using eq. 1 for a cooperative array of activator binding sites (see methods, eq. 13) returns a full model, describing inputs to Hunchback (anterior domain, see also eq S7 and S7a in the Supporting Information File S1):



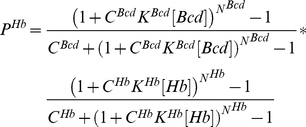
(2)Notice, any other regulatory link, including Bicoid-activator will carry exactly the same Bicoid-specific parameter values (*K^Bcd^*, *C^Bcd^*, *N^Bcd^*). This emulates an assumption that every gene activated by Bicoid carries exactly the same array of Bicoid binding sites. Here and below *p* stands for the maternal (elementary) inputs, *P* for the outputs integrating several maternal inputs. This is true for nearly every other transcriptional regulator (node) in the integrated model. For instance, Hunchback acting as activator or dual regulator utilizes the same set of constants (*K^Hb^*, *C^Hb^*, *N^Hb^*), however, Hunchback acting as a repressor was allowed to use a different set of constants.

***Caudal*** is repressed by Bicoid translationally, however the same framework has been applied to this network connection, given that Bicoid directly binds sites in the *caudal 3’* mRNA (see also eq S8 and S8a in the Supporting Information File S1):



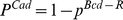
(3)The *caudal* model was a single steady-state model, taking the place of yet another maternal input to the dynamic gap gene network model.

***Kruppel*** is activated and repressed by Hunchback (dual regulation Hb parameters, see also eq S9 and S9a in the Supporting Information File S1) [5]:



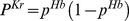
(4)
***Knirps*** is activated by Bicoid and is repressed by Hunchback (Hb-R parameters, see also eq S10 and S10a in the Supporting Information File S1):



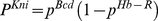
(5)


#### Giant

Either Bicoid or Caudal (operator OR) activate expression of *giant* (see detailed description of the Giant model in the Supporting Information File S1, eq S11 and S11a):



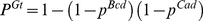
(6)Steady-state models eq. 1, 2, 4, 5 have been described in detail in previous publications [4], [11], [32]. Model eq. 3 fits well the observed distribution of Bicoid and Caudal gradients; model eq. 6 has been developed in this work based on Giant expression in Bicoid and Cad mutants.

### 3. Integrated dynamic model for the gap gene network

The described combination of 6 networks motifs including 4 positional cues (one for each gap gene) and 2 positionally operated toggle switches might represent a minimal architecture, core of the gap gene network. Given that the maternal Bcd gradient is stable in time and the maternal Hb is initial condition for Hb, a quantitative dynamic model for this spatio-temporal network can be expressed using 4 partial differential equations one equation for each trunk gap gene :



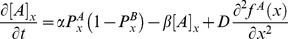
(7)Here, production of a gap gene *A* (Hb, Kr, Kni, Gt) in the spatial coordinate *x* depends on its synthesis rate 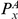 (positional cue) and repression 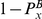 by it's mutual counterpart, repressor *B*. *α*, *β* and *D* here are the synthesis, decay and diffusion rate constants [33], [34] correspondingly. The positional cues and repression terms are expressed via fractional site occupancy models (see methods section) for transcriptional gene networks [4], [35]. Therefore, at the core, the current model is based on gene regulation by transcriptional signals. Transcriptional responses in the system of segmentation genes are mediated by arrays (largely homotypic arrays) of binding sites present in enhancer regions [36], [37], accordingly, every *i* ^th^ connection in the model has been approximated by a response of an *i* ^th^ array of equal binding sites with binding constant *K_i_*, cooperativity of binding *C_i_* and the number of sites *N_i_*. Steady-state models, directly derived from enhancer DNA sequences (distributions of binding sites), recently gained popularity in the fly modeling field [32], [35], [38]. Dynamic models, described here, have all components describing binding site distributions and may be extended to reflect the actual structures of enhancers.

Combining positional cues (*x*-dependent synthesis rates) given by eq. 1–6 with mutual interactions as in eq. 7 returns the following system of four differential equations (eq. 8–11) for the four trunk gap genes:

#### Hunchback

In addition to the positional cues considered above, *hunchback* is repressed by Knirps, substituting the corresponding terms in eq. 7 returns a dynamic model for the anterior Hunchback expression pattern:



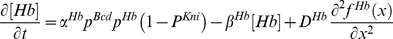
(8)In this model, Knirps repression (1-*P* ^Kni^) is given by eq. 5 with the set of the corresponding Kni - specific parameters, emulating presence of an array of Knirps sites in Hunchback transcription regulatory regions.

***Knirps*** is repressed by Hunchback and Tailless (the former was incorporated in this model as a stable gradient):



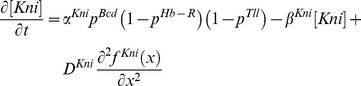
(9)Notice that in the actual model above, the Hunchback repression on Knirps as a positional cue (eq. 5) is indistinguishable from the Hunchback repression on Knirps in the toggle switch. This simply reflects overlapping of the two motifs in the gap network (see **Figure 1**). Repression from Tailless is also incorporated into the Knirps model.

***Kruppel*** is activated and repressed by Hunchback (positional cue) and it is also repressed by Giant:



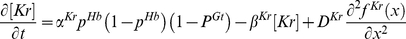
(10)


#### Giant

Either Bicoid or Caudal (positional cue) activate *giant*; Kruppel and Tailless repress *giant*:



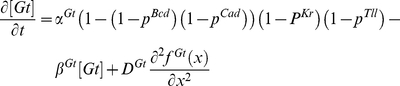
(11)


The system of differential equations (eq. 8–11), describing interactions in the network shown in **Figure 1C** has been explored using standard methods.

### 4. Fitting dynamic model to data using global parameters with realistic values

**Figure 3:**
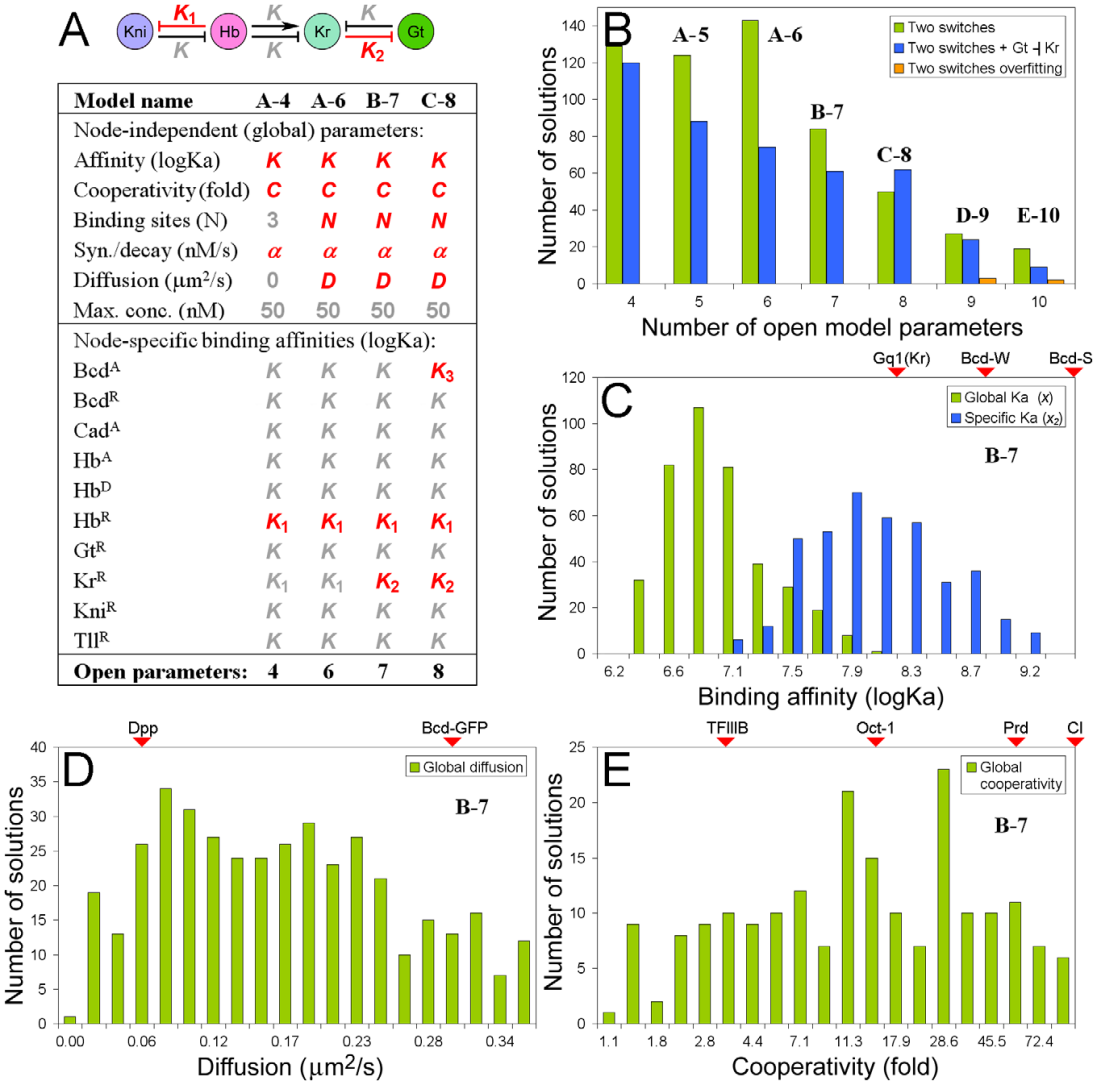
Model design, performance and solution ranges. (A) The number of open for optimization parameters (in red) incorporated into the model. The global parameters included binding affinity, cooperativity, number of binding sites, synthesis, decay and diffusion rates and maximal absolute concentration. Node-specific parameters included binding affinities, different from the global K for some regulatory connections (*K*_1_, *K*_2_, *K*_3_, see the network above). (B) Performance of different models. The explored network with 2 toggle switches performed similarly to the same network plus one link connecting the switches. With more than 8 open parameters, the two-switch model fitted incorrect data (swapped Kr and Kni expression data). (C) Values of global (*K*) and specific (*K*_2_) binding affinity constants from solutions for B-7 model. Markers show known binding affinities for some transcription factors [47], [48]. (D) Values of diffusion for B-7 model. Red triangles on top (markers) show some known diffusion rates [45], [46]. (E) Cooperativity values from solutions for B-7 model, markers show some known cooperativity values [42], [43], [44].

Robust models typically have little or no dependence on the parameter values. If so, a set of global parameters, equal for almost every edge in the gap gene network should still deliver high quality model-data fits. The set of global parameters used in this study is shown in the **Figure 3A**. In all models tested in this study, maximal absolute concentrations [39], maximal synthesis rates, diffusion, decay and cooperativity rates were set equal for all four gap genes (see methods). Preliminary analysis has shown that the condition of a single dynamic attractor (see above) may require unequal repression in the mutual pairs Hb-Kni and Gt-Kr. Given this condition, the binding affinity was set equal (*K*, global) for 12 out of 14 connections (edges) in the network (**Figure 3A**). The toggle switches added 1 (*K*_1_) or 2 (*K*_1_, *K*_2_) node-specific binding affinity parameters to the model (see red network connections in the **Figure 3A**).

Fitting dynamic model (eq. 8–11) to data from FlyEx database [40], [41] demonstrated that even with as few as 4 open parameters (no diffusion [33], fixed number of binding sites, one additional affinity parameter, (see **Figure 3B**) it was possible to obtain multiple high-quality fits (correlation *r*>0.7, see the methods section). However, the best quality fits (*r*>0.9) were obtained for models containing 5–9 parameters, including 3–4 global and 3–5 node-specific (binding affinities *K*, *K*_1_, …*K*_5_) parameters (see also Supporting Information File S1, **Figure S1**). Analysis of solutions, obtained for the 7-parameter model (model “B-7” in **Figure 3A, B**, 4 global, 3 edge-specific: *K*, *K*_1_, *K*_2_) revealed surprisingly realistic parameter values, close to the values observed for transcription factors in this and related systems [42], [43], [44], [45], [46], [47], [48] (see **Figure 3C, D, E**). However, the global binding affinity *K* was off the realistic values in most solutions (**Figure 3D**). This result might be a consequence of the minimalist model design or may suggest that many regulatory interactions in the gap gene network are achieved via vast arrays of relatively weak binding sites [36]. About 50% of all solutions for the model “B-7” returned site arrays containing 3–7 binding sites.

Models with large numbers of parameters may achieve data overfitting, this argument has been raised in many quantitative studies [49]. To detect potential limits for overfitting in this study, models with various number of parameters (4–10) were fit to incorrect data, containing swapped expression data for Kruppel and Knirps. In these fitting tests, successful solutions (*r*>0.7) were detected only for models containing >8 parameters (see **Figure 3B**), however, the number of solutions and their quality were lower. Thus, the main model (7 parameters) is still below the detected overfitting limit.

### 5. Reverse modeling of mutant expression using identified model solutions

Fitting models to data in itself rarely supports any concept, as it simply may be the result of overfitting, typically caused by excessive number of free parameters. Model validation strategies adopted in this study required: first, fitting model to wild type expression data and, second, predicting mutant expression patterns (different data) using parameters, identified at the first, fitting step.

**Figure 4:**
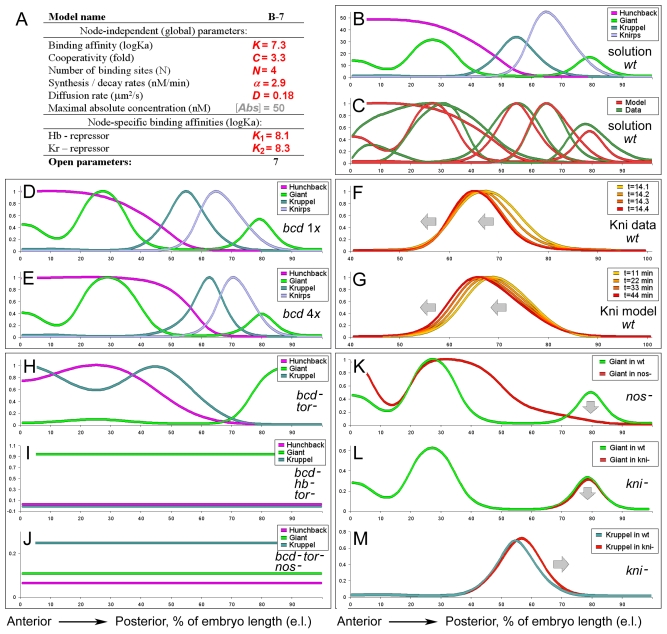
Reverse modeling features of the gap gene network. (A) Parameter values for the analyzed solution. (B) Absolute concentrations (nM) of gap gradients in the selected solution. (C) Data to model agreement (relative concentrations). (D, E) Posterior shift of gap stripes in response to 4x Bicoid. (F, G) Dynamic anterior shift of Knirps stripe. (H–J) Gap gradients in maternal mutants. (H) In the absence of Bcd, *gt* is expressed in the posterior and *Kr* in the anterior. (I) In the absence of both Bicoid and Hunchback, Gt has uniform pattern, while Kr is absent. (J) In the absence of Bicoid, the uniform Hunchback activates *Kr* uniformly; Giant is absent (Gt is low in this simulation). (K–M) Simulation of indirect regulatory links. (K) Uniform maternal Hunchback results in the loss of the posterior Gt stripe. (L) In a *knirps* mutant, the posterior Gt stripe is weakened. (M) Kr stripe in the *knirps* mutant is shifted posteriorly. Model contained no direct links for pairs Hb-Gt, Kni-Gt and Kni-Kr.

Model parameters were constrained based on quantitative expression data (see methods) for the gap genes available from FlyEx database [40]. Best model solutions, matching realistic parameter ranges (**Figure 4A–C**) were examined for dynamics of the stripe patterns and mutant expression. One of the most documented dynamic effects in the system is the anterior shift of gap patterns during cell cycle 14 [6]. The dynamic anterior shift has been reproduced by the model for *kni* and *Kr* expression patterns (**Figure 4F, G**). Dynamics of more terminal Gt stripes was different, perhaps, due to the incomplete set of terminal inputs in the current model. Directly or indirectly, all gap genes are wired to the major *Drosophila* morphogen Bicoid [21]. Increasing the number of copies for *bicoid* gene *in vivo* shifts the entire array of gap and pair-rule stripes to posterior [10]. Simulation of 4 copies of *bcd* reproduced the expected shift for all four trunk gap genes (see **Figure 4D, E**). Mutants for maternal determinants, Bicoid and Hunchback disrupt expression of gap genes [22]; major features of these mutant phenotypes have been reproduced by the model as well (**Figure 4H–J**). Thus, for the *bicoid* null mutant *in vivo* (*bcd* ^-^, *tor* ^-^, to eliminate contribution of terminal system), Kruppel is expressed in the anterior, while Giant is expressed in the posterior. The anterior expression of Kruppel in *bcd* ^-^ is explained by lower concentrations of Hunchback, activating, but not repressing *Kruppel*. This Kruppel expression eliminates the anterior Giant expression (simulation in **Figure 4H**). Removal of Hb from the *bcd* ^-^, *tor* ^-^ embryos eliminates the anterior Kruppel expression, so the Giant is expressed both in the anterior and the posterior regions (simulation in **Figure 4I**). Hb exhibits uniform expression in *bcd* ^-^, *tor* ^-^, *nos* ^-^ resulting in uniform expression of Kruppel and absence of Giant [22]. Though the actual simulation shows residual expression of Giant (see **Figure 4J**), its level is low in comparison to the level of Kr, the ratios Kr:Gt conforms to that observed in this mutant. Along with the mutants for maternal determinants, the model reproduced many mutant phenotypes reflecting direct and indirect regulatory links in the gap gene network [2], [14], [50]. **Figure S2** in Supporting Information File S1 shows simulations for mutants disrupting regulatory interactions in the toggle switches. Typically, removal of one component (Kruppel from Kr-Gt pair) results in a corresponding spatial expansion of its mutual counterpart (Giant, **Figure S2E** in Supporting Information File S1). Successful simulation of these mutants is not too surprising since the corresponding direct links are parts of the model (see **Figure 1C**). It is interesting that the model was able to reproduce some indirect regulatory connections as well.

Consequences of mutations in the Hb-Kni module on the parallel module Gt-Kr are given in **Figure 4K–M**. Despite the absence of direct links between Hb-Gt and Kni-Gt gap pairs, the model was able to correctly reproduce elimination of posterior Gt in *nos* ^-^ mutants expressing uniform Hb (**Figure 4K**) and reduction of posterior Gt stripe in *kni^-^* mutant (**Figure 4L**). Similarly, in the absence of direct Kr-Kni links in the model, the Kr stripe was shifted in the simulated *kni* ^-^ mutant posteriorly, as observed *in vivo* (**Figure 4M**).

Simulated mutant expression using parameters obtained based on the wild type data, has demonstrated reasonable performance of the minimal model proposed for the gap network. Largely, our model produced mutant patterns only at qualitative levels, sometimes with certain aberrant features (**Figure 4J, 4K, 4L**); however, in majority of the considered cases, the qualitative changes in the simulated patterns followed trends observed in the same mutants *in vivo*.

### 6. Mutual repression between the gap genes may be required for the formation of pair-rule stripes

**Figure 5:**
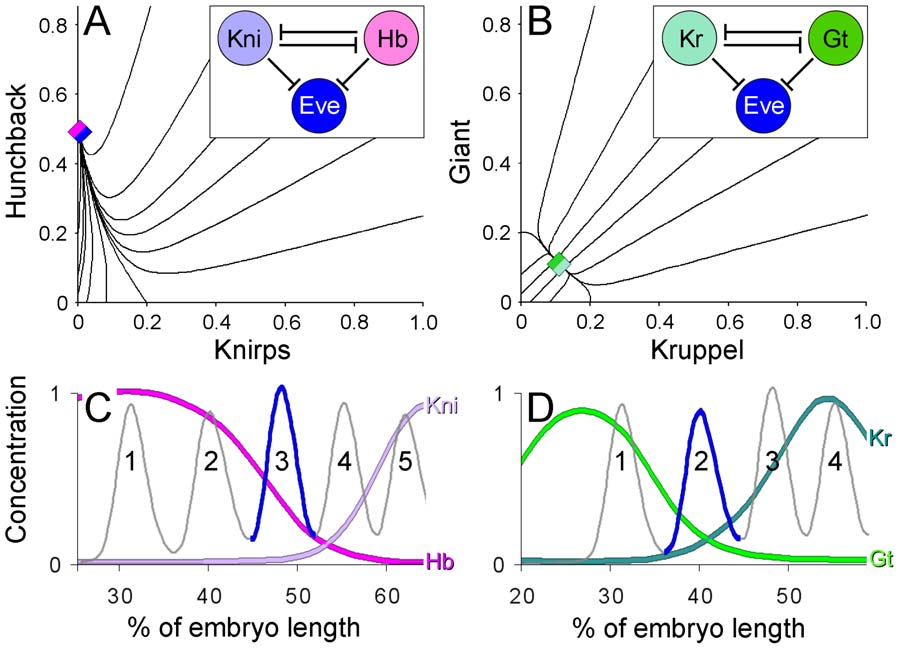
Mutually repressive gradients position pair-rule stripes. (A, B) Phase portraits of monostable toggle switches (as in Figure 2), analogous to gradient pairs establishing expression of Eve stripes (see the network motifs). (C) Formation of the Eve stripe 3 (as well as the Eve stripes 4, 6, 7) requires specific ratio between the Hb and Kni concentrations. (D) Formation of the Eve stripe 2 (as well as the Eve stripe 5) requires specific ratio between the Gt and Kr concentrations. Gradients in (C) and (D) are color-coded in accordance with the network nodes in (A) and (B). Notice, every Eve stripe requires a combination of inputs present in either of the toggle switches (e.g. either Hb+Kni or Gt+Kr).

How do the positional toggle switches operating in the gap gene network affect downstream genes? Concentration ratios between the gap gradients Hb-Kni and Kr-Gt are critical for expression of pair-rule genes, such as *even-skipped* (*eve*) or *hairy* (*h*) [51], [52], [53]. Formation of Eve stripe 2 requires precise ratio between Gt and Kr concentrations (see **Figure 2D, I**); formation of the Eve stripes 3, 4, 6, 7 requires precise ratio of Hb and Kni concentrations [54] (see **Figure 2B, G, H**). Surprisingly, these pairs of gap genes (Gt-Kr, Hb-Kni) correspond to the described toggle switches; no Eve stripes require inputs combining Kni and Gt or Hb and Kr. therefore, the concentration ratios (positional dynamic attractors) established between the gap gradients in response to maternal positional cues may be required to “open windows” for the pair-rule stripes (see **Figure 5**). Without mutual repression, establishing appropriate ratios between the gap gradients would be problematic, thereby compromising the positioning of pair rule stripes.

## Materials and Methods

### Quantitative gene expression data

Quantitative gene expression data were downloaded from the FlyEx database [40]. The input data for the Bicoid and Hunchback gradients corresponded nuclear cleavage cycle 14.1, the output data for Hunchback, Kruppel, Knirps and the input Tailless data corresponded cleavage cycle 14.4. All data was resampled to 100 spatial points (*Δx*  = 5 µm) in the fitting tests.

### Analysis of the toggle switch with variable synthesis rates

To analyze function of the toggle switches, phase portraits were obtained for the following system of ordinary differential equations:
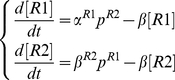
(12)Variable synthesis rates *α* here emulate positional cues of gap genes, changing across the axis coordinate. In all tests, *β* = 0.1, *α* changed in the [0; 1] range and *p* was simulated based on eq. S4 (see Supporting Information File S1) with realistic parameters (see below), equal for both mutual repressors.

### Quantitative framework – binding site occupancy models

For an array of *N* cooperating (*C* - cooperativity fold, 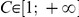) equal binding sites, all with affinity (binding constant) *K*, the probability of occupancy of at least one site in the array is equal to [4], [32]:
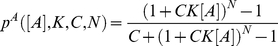
(13)Within this framework, equation 13 is proportional to the probability of activation (rate of synthesis) of a gene, regulated by the transcriptional activator *A*. If *A* is a transcriptional repressor, then the probability of repression of the downstream gene is reverse of *p^A^*. If gene expression is outcome of several regulatory events and they are all required for expression (logical operator “AND”), then the synthesis rate of that gene *P* is given by the product of activation from *i* site arrays for *i* activators and repression from *j* site arrays for *j* repressors as follows [17]:
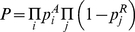
(14)Input integration using logical operator “OR” (multiple independent activators) can be expressed using the following expression:
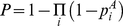
(15)


### Normalization of synthesis rates in the dynamic model

Every dynamic model contained synthesis rates, which include synthesis rate constants *α*, positional cues *p* and mutual repression terms (1-*P*). The synthesis rate constants *α* were equal for all components and were also equal to decay constants *β* (global parameters, see Supporting Information File S1, **Figure S1**). As to maternal positional cues, it has been assumed that the absolute maximal possible rates of synthesis across all spatial coordinates in the embryo, should be similar for all four gap genes at the beginning of their expression. For this reason, values *P*(x) where normalized for every gap gene to [0–1] range for every parameter combination at the initial moment of time. Analytically, this operation is equivalent to incorporation of a multiplier *ω* ^A^ into the synthesis rate terms:
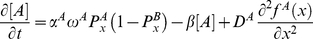
(16)

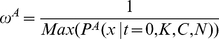
(17)


### Fitting parameters for dynamic model

The Metropolis-Hastings algorithm was used for fitting model to data and obtaining parameter combinations [55], [56]. The objective function of the algorithm was based on the correlation *r*, measured between the model and the data [5]. At every step of the Metropolis algorithm (evaluation of a parameter set), the model-data correlation has been measured for each of the four gap genes, the worst value of this correlation Min(*r*_1_..*r*_4_) has been taken as an argument for the objective function. Solutions for the system of 4 differential equations have been obtained by numerical integration using Euler method (*Δt*  = 2.2 min) for every Metropolis step (Metropolis loop was external to the integration). Diffusion has been simulated by Gaussian filter, applied to the output gradients between every step of the numerical integration (loop, internal to the integration). Probability of acceptance was calculated from the likelihood ratio between the current (*r* ^0^) and the proposed states (*r* ^1^). The proposed state was accepted if the likelihood ratio produced a number greater than a random number *U*, derived from a uniform distribution:
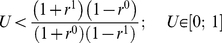
(18)In all fitting tests, the search was run for 500 Metropolis steps per every seed point for 1000 independent seed points in a grid of 100 for every parameter, for exception the number of binding sites (range 1–20). In quality solutions, every gap gene was required to achieve at least 50% (25 nM) of the maximal concentration (50 nM); beyond that, the ratio between the resulting gap gradients was disregarded. Fitting ranges for gap genes were: Hb, 30–70% of embryo length (e.l.); Gt, 10–90% e.l.; Kr, 20–80% e.l.; Kni, 40–90% e.l. All programs used in this study are available in the “Fitting and simulation software” file.

### Simulation of mutant expression

All mutant expression tests *in silico* were carried out based on the same (best) solution with realistic parameter values (the parameters are shown in the **Figure 3** **and Figure S1** in Supporting Information File S1) by removing the corresponding regulatory links from the network and integrating the system of differential equations as described above. Misexpression of the maternal Bicoid and the maternal Hunchback has been simulated by replacing the original maternal gradients in the input data.

## Supporting Information

File S1 The Supporting Information File S1 contains detailed description of fractional occupancy framework, steady-state models used under the synthesis terms and two supplementary figures, Figure S1 describing overfitting test and Figure S2 describing mutant expression.
(PDF)

File S2 The file contains a software package for fitting expression patterns and simulating mutant expression. The software includes help and files for a test run.
(RAR)
