# Supplementary material for: The Drosophila Gap Gene Network Is Composed of Two Parallel Toggle Switches
Source: PLoS One. 2011 Jul 1;6(7):e21145. doi: 10.1371/journal.pone.0021145 (PMC3128594; doi:10.1371/journal.pone.0021145)
Supplement: File S1 — The Supporting Information File S1 contains detailed description of fractional occupancy framework, steady-state models used under the synthesis terms and two supplementary figures, Figure S1 describing overfitting test and Figure S2 describing mutant expression. (PDF) [file pone.0021145.s001.pdf]

The Drosophila gap gene network is composed of two parallel toggle switches

Dmitri Papatsenko and Michael Levine

1. Quantitative framework – binding site occupancy models

Given concentration [*A*] of a transcriptional activator A and a binding affinity *K* of a site for A, probability to occupy this site *p* is equal to:

$$p^A([A],K) = \frac{K[A]}{1+K[A]}; \quad K = e^{\frac{-\Delta G}{RT}} \qquad \text{(S1)}$$

For an array of N equal sites, each with binding affinity constant K, probability that at least one site in that array will be occupied by TF (non-empty states) is equal to:

$$p^A([A],K,N) = \frac{(1+K[A])^N - 1}{(1+K[A])^N} \qquad \text{(S2)}$$

For an array of *N* cooperating ($C = e^{\frac{-\Delta G}{RT}}$, *C* - fold of increased binding) equal binding sites, the probability of occupancy of at least one site is equal to:

$$p^A([A],K,C,N) = \frac{(1+CK[A])^N - 1}{C + (1+CK[A])^N - 1} \qquad \text{(S3)}$$

Within this framework, equation S3 is proportional to the probability of activation (rate of synthesis) of a gene, regulated by the transcriptional activator A. If *R* is a transcriptional repressor, then the probability of repression of the downstream gene is equal to:

$$p^R([R],K,C,N) = 1 - \frac{(1+CK[R])^N - 1}{C + (1+CK[R])^N - 1} = \frac{C}{C + (1+CK[R])^N - 1} \qquad \text{(S4)}$$

If gene expression is outcome of several regulatory events, all required (mode “AND”), then the synthesis rate of that gene *P* is given by the product of activation from *i* site arrays for *i* activators and repression from *j* site arrays for *j* repressors as follows:

$$P = \prod_i p_i^A \prod_j \left(1 - p_j^R\right) \qquad \text{(S5)}$$

If gene expression is the result of *i* activatory events, which supplement each other, but not ultimately required (mode “OR”), then the synthesis rate of the downstream gene is proportional to:

$$P = 1 - \prod_i \left(1 - p_i^A\right) \qquad \text{(S6)}$$

Eq. S1-S6 have been described elsewhere [1,2].

2. Detailed models describing positional cues for maternal and gap genes

***Hunchback*.** Bicoid and Hunchback itself regulate expression of *hunchback*; the both regulators are required (operator AND) for Hunchback expression:

$$P^{Hb} = p^{Bcd} p^{Hb} \tag{S7}$$

Substitution using eq S3 for a cooperative array of activator binding sites returns:

$$P^{Hb} = \frac{\left(1+C^{Bcd}K^{Bcd}[Bcd]\right)^{N^{Bcd}} -1}{C^{Bcd} + \left(1+C^{Bcd}K^{Bcd}[Bcd]\right)^{N^{Bcd}} -1} * \frac{\left(1+C^{Hb}K^{Hb}[Hb]\right)^{N^{Hb}} -1}{C^{Hb} + \left(1+C^{Hb}K^{Hb}[Hb]\right)^{N^{Hb}} -1} \tag{S7a}$$

Notice, any other regulatory link, including Bicoid-activator will carry exactly the same Bicoid-specific parameter values. This emulates an assumption that every gene activated by Bicoid carries exactly the same array of Bicoid binding sites. However, in the case of Bicoid repression (see eq. S8 below) the Bicoid-specific parameters (*K*, *C*, *N*) were allowed to be different (but were the same in the actual models). This is true for every other transcriptional regulator (node) in the integrated model. For instance, Hunchback acting as activator or dual regulator has one sets of constants (*K*, *C*, *N*), Hunchback acting as a repressor was allowed to have different constants. (see Figure 3 in the main text, Figure S1 and eq. S9-S10 below).

Caudal is repressed by Bicoid translationally, however the same framework has been applied to this network connection, given that Bicoid directly binds sites in *caudal* 3' mRNA:

$$P^{Cad} = 1 - p^{Bcd-R} \tag{S8}$$

$$P^{Cad} = \frac{C^{Bcd-R}}{C^{Bcd} + \left(1+C^{Bcd-R}K^{Bcd-R}[Bcd]\right)^{N^{Bcd-R}} -1} \tag{S8a}$$

The *caudal* model was a single steady-state model, taking place of yet another maternal input to the dynamic gap gene network model.

Kruppel is activated and repressed by Hunchback (dual regulation Hb parameters):

$$P^{Kr} = p^{Hb}\left(1 - p^{Hb}\right) \tag{S9}$$

$$P^{Kr} = \frac{\left(1+C^{Hb}K^{Hb}[Hb]\right)^{N^{Hb}} -1}{C^{Hb} + \left(1+C^{Hb}K^{Hb}[Hb]\right)^{N^{Hb}} -1} * \frac{C^{Hb}}{C^{Hb} + \left(1+C^{Hb}K^{Hb}[Hb]\right)^{N^{Hb}} -1} \tag{S9a}$$

Knirps is activated by Bicoid and is repressed by Hunchback (Hb-R parameters):

$$P^{Kni} = p^{Bcd}\left(1 - p^{Hb-R}\right) \tag{S10}$$

$$P^{Kni} = \frac{\left(1 + C^{Bcd} K^{Bcd} [Bcd]\right)^{N^{Bcd}} - 1}{C^{Bcd} + \left(1 + C^{Bcd} K^{Bcd} [Bcd]\right)^{N^{Bcd}} - 1} * \frac{C^{Hb-R}}{C^{Hb-R} + \left(1 + C^{Hb-R} K^{Hb-R} [Hb]\right)^{N^{Hb-R}} - 1} \qquad \text{(S10a)}$$

Giant. Either Bicoid or Caudal (operator OR) activate expression of *giant*:

$$P^{Gt} = 1 - \left(1 - p^{Bcd}\right)\left(1 - p^{Cad}\right) \qquad \text{(S11)}$$

$$P^{Gt} = 1 - \frac{C^{Bcd}}{C^{Bcd} + \left(1 + C^{Bcd} K^{Bcd} [Bcd]\right)^{N^{Bcd}} - 1} * \frac{C^{Cad}}{C^{Cad} + \left(1 + C^{Cad} K^{Cad} [Cad]\right)^{N^{Cad}} - 1} \qquad \text{(S11a)}$$

Models eq. S7, S9, S10 have been described in details in previous publications [1,2,3]. Model eq. S8 fits well the observed distribution of Bicoid and Caudal gradients; model eq. S11 has been developed in this work based on Gt expression in Bicoid and Cad mutants.

3. Model performance and solutions

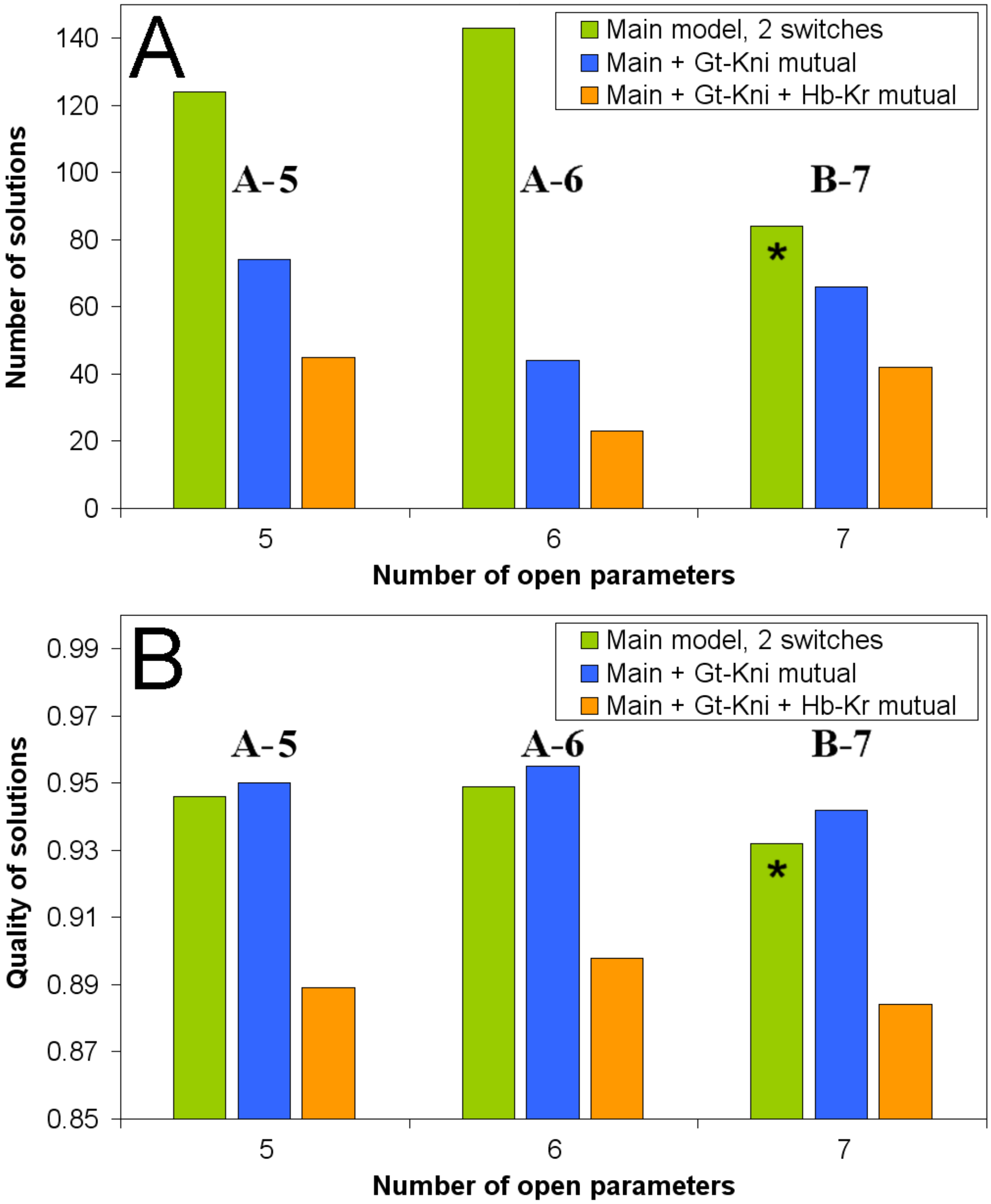

Figure S1. Performance of models with different architecture
(A) Number of solutions and (B) quality of the best solution (correlation, see Methods section) for three different variants (color coded) of the gap gene network. Star marks main model used in this study with 7 parameters open. Incorporation of Gt-Kni mutual repression (in blue) reduces the number of solutions (A), but identifies solutions with better quality (B). Incorporation of both Gt-Kni and Hb-Kr mutual repression (in orange) reduces both the number and the quality of solutions.

4. Model validation

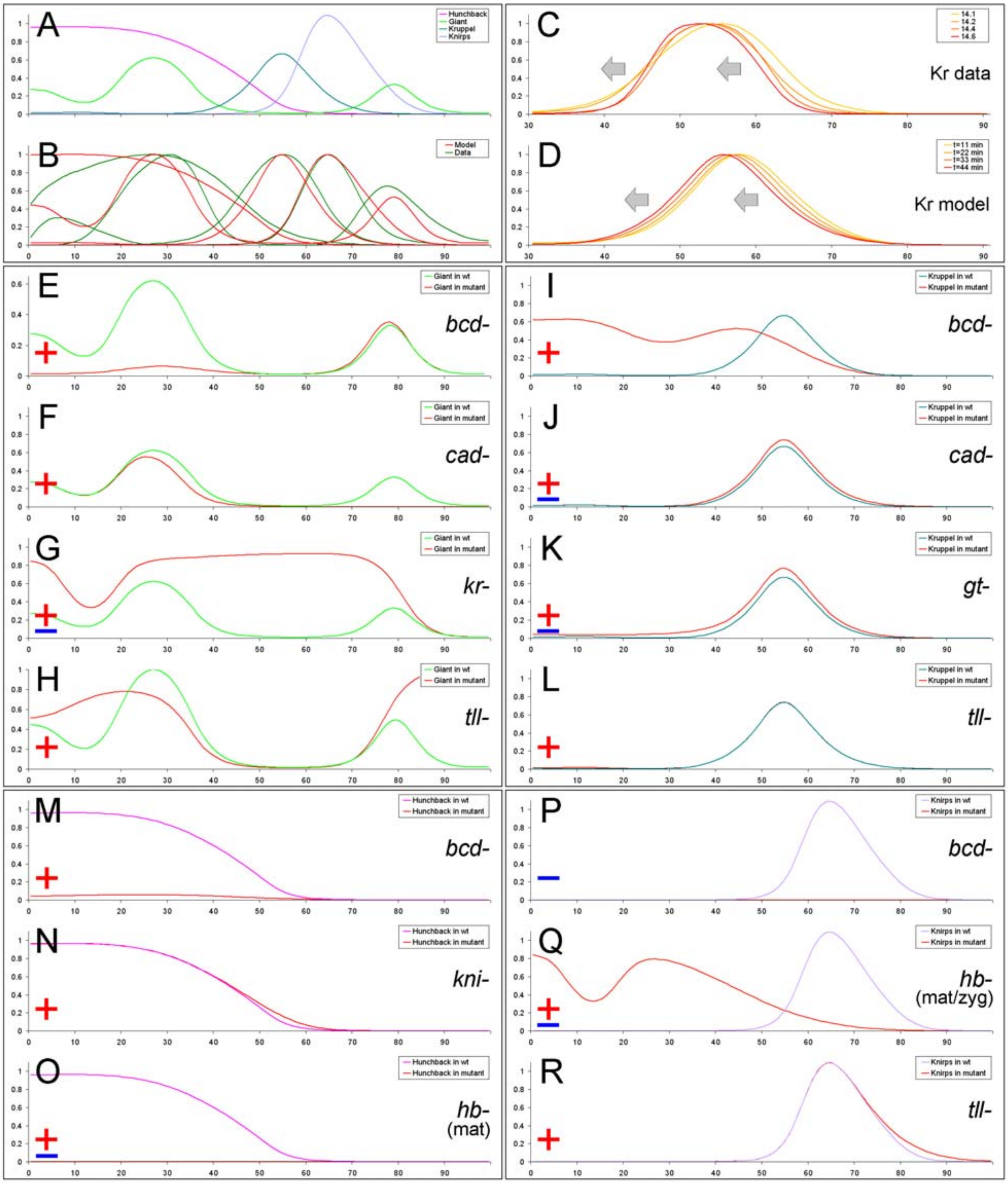

Figure S2. Simulation of mutant expression

(A) Model, (B) model-data fitting, (C-D) anterior shift of Kruppel. (E-H) Simulation of Giant expression in mutants, (I-L) Kruppel expression in mutants, (M-O) Hunchback and (P-R) Knirps expression in mutants. Most mutants, which are not shown (e.g. Hb in Kr^{-}) showed no changes from *wt* in the simulations. Many simulations are in agreement (+) with *in vivo* data [4,5,6,7,8,9,10,11,12,13,14,15,16,17,18]. In the absence of Bicoid, the anterior Giant pattern disappears; Kruppel extends to anterior (A, B); Hunchback anterior and Knirps disappear (I, J). In the absence of Caudal, the posterior Giant pattern disappears (A, C). Zygotic Hunchback disappears in the absence of maternal Hunchback (M). Knirps displays broad anterior expression pattern in the absence of both maternal and zygotic Hunchback (L). In the absence of Knirps, Hunchback anterior patter expands to posterior (K); in the absence of Kruppel, Giant has broad expression, the posterior and the anterior stripes merge (E). Removal of Tailless results in terminal expansion of Giant stripes (G); Knirps posterior stripe (N) and disruption of the central domain Kruppel stripe (H).

References:

1. Zinzen RP, Senger K, Levine M, Papatsenko D (2006) Computational Models for Neurogenic Gene Expression in the Drosophila Embryo. Curr Biol 16: 1358-1365.
2. Zinzen RP, Papatsenko D (2007) Enhancer responses to similarly distributed antagonistic gradients in development. PLoS Comput Biol 3: e84. Epub 2007 Mar 2029.
3. Lopes FJ, Vieira FM, Holloway DM, Bisch PM, Spirov AV (2008) Spatial bistability generates hunchback expression sharpness in the Drosophila embryo. PLoS Comput Biol 4: e1000184.
4. Driever W, Nusslein-Volhard C (1989) The bicoid protein is a positive regulator of hunchback transcription in the early Drosophila embryo. Nature 337: 138-143.
5. Struhl G, Struhl K, Macdonald PM (1989) The gradient morphogen bicoid is a concentration-dependent transcriptional activator. Cell 57: 1259-1273.
6. Hulskamp M, Schroder C, Pfeifle C, Jackle H, Tautz D (1989) Posterior segmentation of the Drosophila embryo in the absence of a maternal posterior organizer gene. Nature 338: 629-632.
7. Hulskamp M, Pfeifle C, Tautz D (1990) A morphogenetic gradient of hunchback protein organizes the expression of the gap genes Kruppel and knirps in the early Drosophila embryo. Nature 346: 577-580.
8. Kraut R, Levine M (1991) Spatial regulation of the gap gene giant during Drosophila development. Development 111: 601-609.
9. Kraut R, Levine M (1991) Mutually repressive interactions between the gap genes giant and Kruppel define middle body regions of the Drosophila embryo. Development 111: 611-621.
10. Hulskamp M, Tautz D (1991) Gap genes and gradients--the logic behind the gaps. Bioessays 13: 261-268.
11. Eldon ED, Pirrotta V (1991) Interactions of the Drosophila gap gene giant with maternal and zygotic pattern-forming genes. Development 111: 367-378.
12. Struhl G, Johnston P, Lawrence PA (1992) Control of Drosophila body pattern by the hunchback morphogen gradient. Cell 69: 237-249.
13. Hoch M, Gerwin N, Taubert H, Jackle H (1992) Competition for overlapping sites in the regulatory region of the Drosophila gene Kruppel. Science 256: 94-97.
14. Capovilla M, Eldon ED, Pirrotta V (1992) The giant gene of Drosophila encodes a b-ZIP DNA-binding protein that regulates the expression of other segmentation gap genes. Development 114: 99-112.
15. Simpson-Brose M, Treisman J, Desplan C (1994) Synergy between the hunchback and bicoid morphogens is required for anterior patterning in Drosophila. Cell 78: 855-865.
16. Hulskamp M, Lukowitz W, Beermann A, Glaser G, Tautz D (1994) Differential regulation of target genes by different alleles of the segmentation gene hunchback in Drosophila. Genetics 138: 125-134.
17. Rivera-Pomar R, Lu X, Perrimon N, Taubert H, Jackle H (1995) Activation of posterior gap gene expression in the Drosophila blastoderm. Nature 376: 253-256.
18. Rivera-Pomar R, Jackle H (1996) From gradients to stripes in Drosophila embryogenesis: filling in the gaps. Trends Genet 12: 478-483.
